# Risk of depressive and anxiety disorders in young adults with disabilities: a nationwide cohort study in South Korea

**DOI:** 10.1017/S204579602510036X

**Published:** 2026-01-05

**Authors:** Hwa-Young Lee, In Young Cho, Dong Wook Shin, Kyung-Do Han

**Affiliations:** 1 Graduate School of Public Health and Healthcare Management, The Catholic University of Korea, Seoul, Republic of Korea; 2 Catholic Institute for Public Health and Healthcare Management, The Catholic University of Korea, Seoul, Republic of Korea; 3 Department of Family Medicine and Supportive Care Center, Samsung Medical Center, Sungkyunkwan University School of Medicine, Seoul, Republic of Korea; 4 Department of Clinical Research Design and Evaluation, Samsung Advanced Institute for Health Sciences and Technology (SAIHST), Sungkyunkwan University, Seoul, Republic of Korea; 5 Department of Statistics and Actuarial Science, Soongsil University, Seoul, Republic of Korea

**Keywords:** common mental disorders, epidemiology, social factors, survival analysis, young adults

## Abstract

**Background:**

Although often associated with ageing, disability is becoming increasingly prevalent among young adults. While disability can pose a substantial psychological burden for young adults on critical pathways to establish the foundations for their future, the mental health risks faced by this population remain underexplored.

**Aims:**

This study aimed to (1) assess the association between disability – including its presence, severity and type – and the risk of depressive and anxiety disorders, and (2) examine whether this association varies across sociodemographic factors, health behaviours and comorbidities in a young adult population.

**Methods:**

We conducted a population-based cohort study using linked data from the National Disability Registry and the National Health Insurance Database of South Korea. A total of 6,058,290 individuals aged 20–39 years who underwent health check-ups between 2009 and 2012 were followed through 2022. Cox proportional hazards models were used to estimate adjusted hazard ratios (aHRs) for depressive and anxiety disorders.

**Results:**

Individuals with disabilities had significantly higher risks of depressive (aHR: 1.58, 95% CI: 1.55–1.60) and anxiety disorders (aHR: 1.50, 95% CI: 1.42–1.59). Increased risks were consistently observed across various disability types with the highest risk observed for mental health-related disabilities in depression (aHR: 4.98, 95% CI 4.62–5.37) and epilepsy-related disabilities in anxiety disorders (aHR: 12.05, 95% CI 8.73–16.63). Subgroup analyses revealed stronger associations among individuals in their 20s, low-income groups, non-smokers and those abstaining from alcohol, compared to their respective counterparts.

**Conclusions:**

Young adults with disabilities, a population that has been relatively overlooked in policy discussions, warrant greater policy attention in relation to their mental health.

## Introduction

Globally, over 1.3 billion people – approximately one in six – live with some form of disability, and this number continues to grow, driven largely by population ageing and the rising burden of non-communicable diseases (NCDs) (Organization, 2022). Although disability is closely linked to ageing, its prevalence is also markedly increasing among young adults. In the US, for example, the proportion of adults aged 18 to 44 reporting at least one disability or activity limitation rose by five percentage points between 2000 and 2018 (Zajacova and Margolis, 2024). Similarly, recent data from Canada show that disability prevalence among individuals increased most sharply among younger populations – by seven percentage points among those aged 15–24 years and by four percentage points among those aged 25–64 years – compared to a three-point increase among older adults (≥65 years) (Canada, December 1, 2023).

There is substantial evidence linking disability to increased risk of mental health conditions, and mental health-related mortality, including dementia (Cho *et al.*, 2023), anxiety, depression (Kwon *et al.*, 2024) and suicide mortality (Lee *et al.*, 2024). These studies have consistently reported that the impact of disability on mental comorbidities is more pronounced among younger age groups than older ones (Cho *et al.*, 2023; Lee *et al.*, 2024), underscoring the importance of early identification and prevention of these psychiatric conditions beginning in early adulthood.

Despite these implications, research and policy discussions have disproportionately focused on the elderly or children, leaving young adults relatively overlooked. Young adulthood in their 20s and 30s represents a pivotal life stage characterised by transition towards independence, decision-making responsibility, as well as the building of competencies and human capital, thereby setting the trajectory for career development, socioeconomic stability and family formation later in life (Pinquart, 2014). The onset of disability during this formative period may mean substantial loss of opportunities, leading to cumulative disadvantage across the life course, and therefore, can lead to greater frustration and lower self-esteem, making young adults more vulnerable to mental health disorders, compared to the older population (Kalin, 2020).

Anxiety and depressive disorders are among the most common psychiatric illnesses linked to impaired quality of life (Hansson, 2002) and suicide (Kalin, 2021), and frequently co-occur (Kalin, 2020). Although several studies have examined the relationship between disability and these mental health conditions among non-elderly, most have focused on children and adolescents under 20 years or only in their early 20s (Botting *et al.*, 2016; Dykens *et al.*, 2015; Lal *et al.*, 2022; Maiano *et al.*, 2018; Strang *et al.*, 2012), or on specific types of disability such as intellectual (Bakken *et al.*, 2010; Dykens *et al.*, 2015; lugnegård *et al.*, 2011; Strang *et al.*, 2012), physical (Verhoof *et al.*, 2013), or language impairments (Botting *et al.*, 2016). In addition, many of these studies employed relatively small sample sizes, consisting of only 100 ∼ 200 participants (Bakken *et al.*, 2010; Botting *et al.*, 2016; Dykens *et al.*, 2015; lugnegård *et al.*, 2011).

To our knowledge, no prior studies have comprehensively assessed the risk for depressive and anxiety disorders among young adults aged 20–39 years with disabilities in a large, representative sample. This study, therefore, aims to: 1) examine the association between disability – including its presence, severity and type – and the risk of anxiety and depressive disorder, and 2) explore the heterogeneity of this association across sociodemographic factors, health behaviours and comorbid conditions.

## Methods

### Data sources

This study utilised two population-based datasets from the Republic of Korea: the National Health Insurance (NHI) Database and the National Disability Registry (NDR). Korea operates a single-payer public health insurance system that covers approximately 97% of the population, funded through enrollees’ premium contributions. The remaining 3% – those living below the poverty line – are supported through the Medical Aid (MA) scheme, which is fully funded by general taxation. The NHI Database comprises several sub-datasets, of which we utilised the following three: (1) the NHI Enrollee Database, which provides basic sociodemographic information on enrollees; (2) the Claims Database, which includes detailed records of disease diagnoses and health service utilisation; and (3) the Health Check-up Database, which offers information on health-related behaviours (e.g., smoking status and alcohol consumption), anthropometric measurements (e.g., body mass index [BMI]) and laboratory biomarkers such as blood pressure, fasting glucose and lipid profiles (Shin *et al.*, 2022). The NDR contains information on individuals with officially registered disabilities, including disability type and severity levels, as determined by standardised clinical assessment protocols. The National Health Insurance Service (NHIS) offers researchers access to a dataset that links the NHI data with selected information from NDR for research purposes. Further details on each data source are provided in the Supplementary Methods.

### Study population and design

Our study population was derived from the Health Check-up Database, which includes individuals who participated in the biennial health check-up program provided by the NHI agency. The initial, nationally representative sample comprised 6,891,401 individuals aged 20–39 years, including both individuals with and without disabilities who completed the Health Check-up between 2009 and 2012. This sample represents approximately 40% of the population within the specified age group. Observations with missing values in any of the study variables were excluded, resulting in a refined sample of 6,328,087 individuals. Additionally, individuals with pre-existing diagnoses of depressive or anxiety disorders at baseline were excluded to ensure that only incident cases were analysed. A one-year lag was applied to the outcome variable to address potential temporal effects and avoid the risk of reverse causality. The final analytical cohort consisted of 6,058,290 individuals, including 5,970,401 without disabilities and 87,889 with disabilities.

### Outcome variable and follow-up

The outcome variables were the incidence of depressive and anxiety disorders. These conditions were identified using the diagnostic codes from the Korean Classification of Diseases, 6th revision (KCD-6), within the NHI Claims Database. KCD-6 is an extended version of the ICD-10, incorporating disease classifications used in traditional Korean medicine (Ko *et al.*, 2020).

The incidence of depressive and anxiety disorders was defined as having either two or more outpatient visits or at least one hospitalisation with relevant diagnostic codes (F32.x and F33.x for depressive disorder, and F40.x and F41.x for anxiety disorder) (Kwon *et al.*, 2024). Each individual was followed from the date of their health check-up until the occurrence of depressive or anxiety disorder, emigration, or December 31, 2022 – whichever occurred first.

### Independent variables

The primary independent variables of interest were the presence of the disability, as well as its severity and types. Disability severity within the NDR is classified into six grades based on the degree of functional impairment, which is quantified as a percentage of functional loss relative to pre-impairment functionality. Specifically, Grade 1 (most severe) indicates a functional loss exceeding 85%; Grade 2, 75–84%; Grade 3, 60–74%; Grade 4, 45–59%; Grade 5, 35–44%; and Grade 6 (mildest), 25–34%. These were dichotomised into Grades 1–3 (severe) and Grades 4–6 (mild). The specific functional domains assessed for severity classification vary by disability type (Kim *et al*., 2023). To ensure objectivity and inter-rater consistency, the assessment process follows two-step procedures based on national criteria. First, a specialist at an approved medical institution conducts the initial assessment. The results are then submitted to the National Pension Service, where a medical advisory committee – comprising at least two specialists – reviews the case and renders the final decision (MoHW). Further details on the Korean NDR are available in Kim et al. (2023). Disability types are classified into 15 categories within the NDR, in accordance with the classification framework defined by the Korean Welfare Act for Persons with Disabilities.

Covariates included sociodemographic characteristics (sex, age and income level), health-related behaviours (smoking status, alcohol consumption and regular physical activity) and comorbidities (obesity, metabolic syndrome, diabetes mellitus [DM], hypertension, dyslipidemia). As the NHI Database does not provide specific household income information, monthly health insurance premiums were used as a proxy for income. The NHI premiums are determined based on gross salary for employee-insured individuals and on a composite index of annual income and assets for self-employed enrollees. Low-income was defined as a combination of MA beneficiaries and the 1st quartile of NHI enrollees. More specific definitions and operational criteria for income and comorbidities are provided in Table S1.

### Analyses

Descriptive statistics were presented for the study sample, stratified by disability status and severity. The cumulative incidence of depressive and anxiety disorders was illustrated using Kaplan–Meier survival curves, stratified by disability status and severity, to visualise differences in time-to-event distributions. To examine the association between disability and the incidence of depressive and anxiety disorders, Cox proportional hazards regression models were employed. Three primary models were constructed: Model 1 (M1) assessed the presence of disabilities; Model 2 (M2) examined the dichotomised categories of disability severity; and Model 3 (M3) investigated disability types. Hazard ratios (HRs) and corresponding 95% confidence intervals (CIs) were estimated, with statistical significance determined by a two-sided P-value of < 0.05. For each model, we sequentially adjusted for two blocks of covariates to examine the potential confounding effect: first adjusting for demographic variables (age and sex), and then additionally adjusting for economic status, health-related behaviours and comorbidities.

In addition, comprehensive subgroup analyses were conducted to explore potential heterogeneity in the associations between disability and the incidence of depressive and anxiety disorders, across strata defined by sociodemographic characteristics, health behaviours and comorbidity profiles.

## Results

### Characteristics of the study sample

A total of 87,889 individuals, accounting for 1.45% of the study population, were identified as having a disability (Table 1). Among them, approximately 33.8% were classified as having a severe disability, defined as Grades 1 to 3. The most prevalent type of disability was related to the extremities, comprising 53.8% of the disabled group, followed by visual (12.8%) and intellectual disabilities (12.3%). The mean age was higher in the disability group than in the non-disability group (32.3 years vs. 30.8 years). Additionally, the proportion of individuals classified as low-income was greater among those with disability compared to those without (Table 1).

**Table 1.** Sample characteristics by disability status: n (%)

			Total	Without disability	With disability	
Categories			6,058,290	5,970,401 (98.5)	87,889 (1.5)	P-value
Disability	Disability severity	Mild (Grades 4–6)			58,185 (66.2)	
		Severe (Grades 1–3)			29,704 (33.8)	
	Disability type	Intellectual disability			10,832 (12.3)	
		Disability due to autism			379 (0.4)	
		Disability due to mental disorders			1,544 (1.8)	
		Disability due to brain impairment			2,705 (3.1)	
		Disability due to epilepsy			366 (0.4)	
		Disability due to renal failure			1,169 (1.3)	
		Disability due to heart conditions			230 (0.3)	
		Disability due to ostomy			215 (0.2)	
		Disability due to liver disease			85 (0.1)	
		Disability due to respiratory conditions			60 (0.1)	
		Facial deformity disability			306 (0.3)	
		Disabilities in extremities			51,198 (53.8)	
		Visual disability			11,233 (12.8)	
		Hearing disability			6,586 (7.5)	
		Speech and language disability			981 (1.1)	
Sociodemographic characteristics	Sex	Male	3,626,309 (59.9)	3,555,048 (59.5)	71,261 (81.1)	<.0001
	Age (mean ± SD*)		30.8 ± 5.0	30.8 ± 5.0	32.3 ± 5.0	<.0001
	Age group	20–29	2567062 (42.4)	2,542,000 (42.6)	25,062 (28.5)	<.0001
		30–39	3491228 (57.6)	3,428,401 (57.4)	62,827 (71.5)	
	Income level	Low	1,287,707 (21.3)	1,258,148 (21.1)	29,559 (33.6)	<.0001
Health-related behavior	Currently smoking		2,128,594 (35.1)	2,092,237 (35.0)	36,357 (41.4)	<.0001
	Currently drinking alcohol		3,794,495 (62.6)	3,744,189 (62.7)	50,306 (57.2)	<.0001
	Currently regular exercise		778,261 (12.9)	764,459 (12.8)	13,802 (15.7)	<.0001
Comorbidities	Obesity		1,612,896 (26.6)	1,580,978 (26.5)	31,918 (36.3)	<.0001
	Metabolic syndrome		648,978 (10.7)	633,759 (10.6)	15,219 (17.3)	<.0001
	Diabetes		116,283 (1.9)	112,975 (1.9)	3,308 (3.6)	<.0001
	Hypertension		454,775 (7.5)	443,307 (7.4)	11,468 (13.1)	<.0001
	Dyslipidemia		410,202 (6.8)	402,056 (6.7)	8,146 (9.3)	<.0001
Biometrics	Height, cm		168.3 ± 8.4	168.3 ± 8.3	169.2 ± 9.2	<.0001
	Weight, kg		65.6 ± 13.6	65.6 ± 13.6	68.9 ± 14.1	<.0001
	BMI, kg/m^2^		23.0 ± 3.6	23 ± 3.6	23.9 ± 3.9	<.0001
	Waist Circumference, cm		77.6 ± 10.0	77.5 ± 10.0	81.0 ± 9.9	<.0001
	Fasting glucose, mg/dL		90.9 ± 16.6	90.8 ± 16.5	93.4 ± 21.0	<.0001
	Systolic BP, mmHg		117.8 ± 13.2	117.8 ± 13.2	120.8 ± 13.6	<.0001
	Diastolic BP, mmHg		73.8 ± 9.5	73.8 ± 9.45	75.9 ± 9.75	<.0001
	Total Cholesterol, mg/dL		184.6 ± 33.8	184.5 ± 33.7	186.6 ± 35.7	<.0001
	HDL-C, mg/dL		57.3 ± 21.9	57.4 ± 21.9	54 ± 20.4	<.0001
	LDL-C, mg/dL		104.8 ± 34.4	104.7 ± 34.4	106.4 ± 35.8	<.0001
	Triglyceride, mg/dL		96.8 (96.7–96.8)	96.6 (96.5–96.6)	111.5 (111.1–112.0)	<.0001
Outcome						
Incidence of outcome	Depression		730,330 (12.1)	715,465 (12.0)	14,865 (16.9)	<.0001
	Anxiety		60,624 (1.0)	59,391 (1.0)	1,233 (1.4)	<.0001
Mean ± SD	Depression		10.7 ± 2.4	10.7 ± 2.4	10.4 ± 2.8	<.0001
	Anxiety		11.3 ± 1.3	11.3 ± 1.3	11.2 ± 1.5	<.0001
Median (Q1–Q3)	Depression		11.4 (10.1–12.2)	11.4 (10.1–12.2)	11.3 (9.7–12.2)	<.0001
	Anxiety		11.6 (10.5–12.4)	11.6 (10.5–12.2)	11.7 (10.4–12.2)	<.0001

Health-related behaviours and comorbidity profiles also differed by disability status. Compared to those without disabilities, individuals with disabilities had higher smoking rates but lower rates of alcohol consumption and higher rate of engagement in regular exercise. Comorbidities such as obesity and metabolic syndrome were more prevalent in the disability group. Overall, the incidence of depressive disorder was higher than that of anxiety disorder, with both conditions occurring more frequently among individuals with disability than those without (Table 1).

Within the disability group, those with severe disabilities were more likely to be younger, have low income, and less likely to smoke or consume alcohol or have comorbidities. Depression and anxiety disorders occurred at a higher rate among individuals with severe disabilities compared to those with mild disabilities (Table S2).

### Risk of depressive and anxiety disorders by disability status

**Figure 1.**
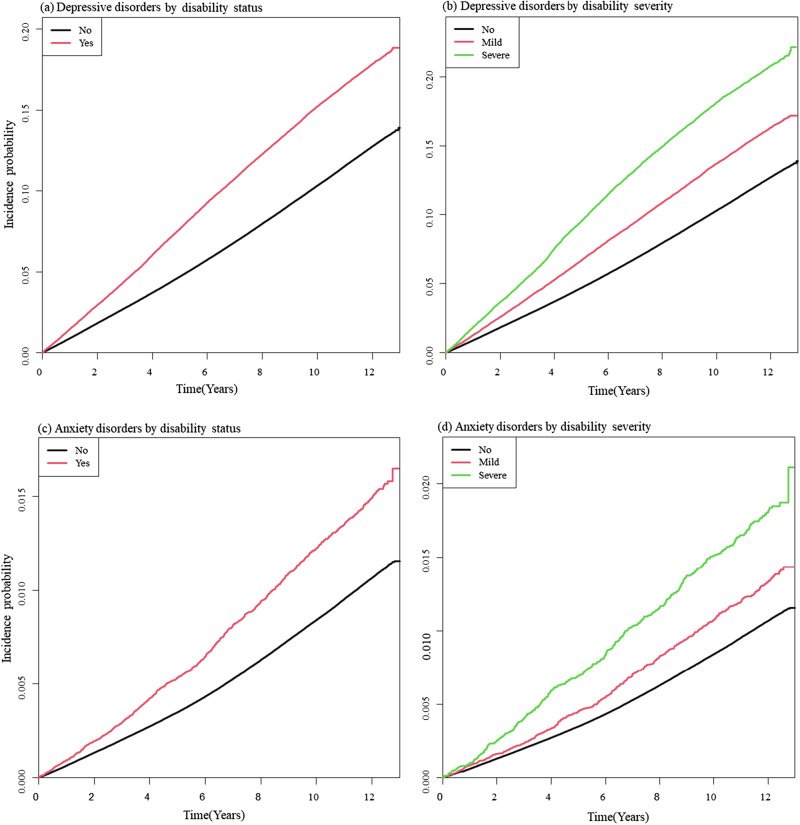
(a–d) Kaplan–Meier curves illustrating the cumulative incidence of depressive and anxiety disorders by disability status and severity over time.

The cumulative incidence of both depressive and anxiety disorders was higher among individuals with disabilities (Fig. 1(a) and (c)), with those with severe disabilities exhibiting a higher cumulative incidence than those with mild disabilities (Fig. 1(b) and (d)). When fully adjusted for covariates, individuals with disabilities were 1.58 times (95% CI: 1.55–1.60) more likely to develop depressive disorders and 1.50 times (95% CI: 1.42–1.59) more likely to develop anxiety disorders compared to those without disabilities (Table 2). The increased risk was more pronounced for severe disabilities (adjusted HR [aHR] [95% CI]: 1.88 [1.84–1.93] for depressive disorder; 1.87 [1.71–2.04] for anxiety disorder) than mild disabilities (aHR [95% CI] = 1.42 [1.39–1.45] for depressive disorder; 1.32 [1.23–1.42] for anxiety disorder) (Table 2).

**Table 2.** Association between disability and incidence of depressive and anxiety disorders

			Depression								Anxiety							
Categories^a^		Total N	n	Person-years^c^	Non-adjusted			Fully adjusted^d^			n	Person-years^b^	Non-adjusted			Fully adjusted^d^		
Disability status	Without disability	5,970,401	715,465	64,047,206	1(Ref.)			1 (Ref.)			59,391	67,539,014	1 (Ref.)			1 (Ref.)		
	With disability	87,889	14,865	909,574	1.47	(1.44,	1.49)	1.58	(1.55,	1.60)	1,233	986,853	1.43	(1.35,	1.51)	1.50	(1.42,	1.59)
Disability severity	No disability	5,970,401	715,465	64,047,206	1 (Ref.)			1 (Ref.)			59,391	67,539,014		1 (Ref.)			1 (Ref.)	
	Mild (Grades 4–6)	58,185	9,041	613,141	1.32	(1.29,	1.35)	1.42	(1.39,	1.45)	736	659,928	1.27	(1.18,	1.36)	1.32	(1.23,	1.42)
	Severe (Grades 1–3)	29,704	5,824	296,433	1.77	(1.73,	1.82)	1.88	(1.84,	1.93)	497	326,925	1.74	(1.59,	1.90)	1.87	(1.71,	2.04)
Disability type	No disability	5,970,401	715,465	64,047,206	1 (Ref.)			1 (Ref.)			59,391	67,539,014	1 (Ref.)			1 (Ref.)		
	Intellectual disability	10,832	2,175	105,661	1.87	(1.79,	1.95)	2.05	(1.96,	2.14)	195	116,658	1.92	(1.67,	2.21)	2.18	(1.89,	2.51)
	Disability due to autism	379	70	3,651	1.74	(1.38,	2.20)	2.27	(1.79,	2.87)	2	4,049	0.57	(0.14,	2.28)	0.73	(0.18,	2.92)
	Disability due to mental disorders^b^	1,544	679	12,446	5.06	(4.69,	5.45)	4.98	(4.62,	5.37)	44	16,320	3.11	(2.32,	4.18)	3.26	(2.42,	4.38)
	Disability due to brain damage	2,705	561	26,743	1.89	(1.74,	2.06)	2.02	(1.86,	2.19)	64	29,691	2.47	(1.93,	3.15)	2.62	(2.05,	3.35)
	Disability due to epilepsy	366	128	3,231	3.61	(3.04,	4.30)	3.82	(3.21,	4.54)	37	3,778	11.27	(8.17,	15.56)	12.05	(8.73,	16.63)
	Disability due to renal failure	1,169	198	11,684	1.53	(1.33,	1.76)	1.57	(1.36,	1.80)	27	12,696	2.44	(1.67,	3.55)	2.48	(1.70,	3.61)
	Disability due to heart conditions	230	41	2,287	1.62	(1.19,	2.20)	1.68	(1.24,	2.28)	2	2,513	0.91	(0.23,	3.64)	0.94	(0.23,	3.75)
	Disability due to ostomy	215	38	2,060	1.68	(1.22,	2.31)	1.85	(1.34,	2.54)	1	2,253	0.52	(0.07,	3.65)	0.55	(0.08,	3.87)
	Disability due to liver disease	85	14	849	1.49	(0.88,	2.51)	1.56	(0.93,	2.64)	2	916	2.51	(0.63,	10.02)	2.66	(0.67,	10.63)
	Disability due to respiratory conditions	60	10	604	1.50	(0.81,	2.78)	1.54	(0.83,	2.86)	0	646	.			.		
	Facial deformity disability	306	52	3,195	1.46	(1.11,	1.92)	1.54	(1.17,	2.02)	3	3,446	0.99	(0.32,	3.08)	1.03	(0.33,	3.20)
	Disabilities in extremities	51,198	8130	538,771	1.35	(1.32,	1.38)	1.45	(1.42,	1.49)	653	581,312	1.28	(1.18,	1.38)	1.33	(1.23,	1.44)
	Visual disability	11,233	1636	118,464	1.24	(1.18,	1.30)	1.33	(1.27,	1.39)	119	126,523	1.07	(0.89,	1.28)	1.12	(0.93,	1.34)
	Hearing disability	6,586	973	69,703	1.25	(1.17,	1.33)	1.30	(1.22,	1.38)	63	750,020	0.95	(0.74,	1.22)	0.99	(0.77,	1.26)
	Speech and language disability	981	160	10,224	1.40	(1.20,	1.64)	1.54	(1.32,	1.79)	21	11,033	2.16	(1.41,	3.32)	2.33	(1.52,	3.57)

a Separate analyses were conducted for disability status severity and type.

b Mental disabilities other than those due to depressive or anxiety disorders

c Duration of follow-up

d Adjusted for sex, age, income level, smoking, drinking alcohol, physical activities, obesity, diabetes mellitus, hypertension, dyslipidemia

Patterns of association by disability type differed between the two mental health conditions. The greatest increase in risk for depressive disorder was observed among individuals with mental disorder-related disabilities (aHR [95% CI] = 4.98 [4.62–5.37]). On the other hand, epilepsy-related disability was associated with the highest risk for anxiety disorder, with an approximately 12.1-fold increase compared to individuals without disabilities, showing a substantial gap from the second-highest risk category (aHR [95% CI] = 3.26 [2.42–4.38] for disability due to mental disorders). Several disability types – such as those related to autism, ostomy and heart conditions, facial deformities and visual and hearing impairments – were associated with an increased risk of depressive disorder but not with anxiety disorder incidence (Table 2). Adjustment for demographic characteristics slightly increased the HRs, whereas additional adjustment of economic status, health-related behaviour and comorbidities had little influence on the observed associations (Table S2).

### Stratified analyses by sociodemographic and health-related characteristics

Several factors were common effect modifiers of the association between disability and the incidence of both depressive and anxiety disorders (Table 3). The HRs for both mental health outcomes associated with disability were significantly higher among individuals aged 20–29 years compared to those aged 30–39 years (aHR [95% CI] = 1.64 [1.59–1.69] vs. 1.56 [1.35–1.59], p-interaction = 0.006 for depressive disorder) and in the low-income group compared to the higher-income group (aHR [95% CI] = 1.86 [1.81–1.91] vs. 1.44 [1.41–1.47], p-interaction < 0.001 for depressive disorder; 1.87 [1.70–2.05] vs. 1.33 [1.24–1.43], p-interaction < 0.001 for anxiety disorder). The association was also more pronounced among individuals who did not smoke (aHR [95% CI] = 1.67 [1.64–1.71] vs. 1.44 [1.40–1.47], p-interaction < 0.001 for depressive disorder; 1.71 [1.59–1.83] vs. 1.22 [1.11–1.34], p-interaction < 0.001 for anxiety disorder), did not drink alcohol (aHR [95% CI] = 1.77 [1.73–1.81] vs. 1.43 [1.40–1.46], p-interaction < 0.001 for depressive disorder; 1.78 [1.64–1.93] vs. 1.30 [1.20–1.40], p-interaction < 0.001 for anxiety disorder), compared to their respective counterparts.

**Table 3.** Association between disability and incidence of depressive and anxiety disorders: stratified analyses by sociodemographic and health-related factors

		Depressive disorders: HR (95% CI)					Anxiety disorders: HR (95% CI)				
Variables	Category	Without disability	With disability				Without disability	With disability			P for interaction
Sex	Male	1 (Ref)	1.55	(1.53,	1.58)	0.007	1 (Ref)	1.46	(1.37,	1.56)	0.091
	Female	1 (Ref)	1.64	(1.58,	1.69)		1 (Ref)	1.63	(1.46,	1.83)	
Age (years)	20–29	1 (Ref)	1.64	(1.59,	1.69)	0.006	1 (Ref)	1.67	(1.51,	1.86)	0.015
	30–39	1 (Ref)	1.56	(1.53,	1.59)		1 (Ref)	1.44	(1.34,	1.54)	
Income	Higher	1 (Ref)	1.44	(1.41,	1.47)	< .0001	1 (Ref)	1.33	(1.24,	1.43)	< .0001
	Low	1 (Ref)	1.86	(1.81,	1.91)		1 (Ref)	1.87	(1.70,	2.05)	
Current smoking	No	1 (Ref)	1.67	(1.64,	1.71)	< .0001	1 (Ref)	1.71	(1.59,	1.83)	< .0001
	Yes	1 (Ref)	1.44	(1.40,	1.47)		1 (Ref)	1.22	(1.11,	1.34)	
Drinking	No	1 (Ref)	1.77	(1.73,	1.81)	< .0001	1 (Ref)	1.78	(1.64,	1.93)	< .0001
	Yes	1 (Ref)	1.43	(1.40,	1.46)		1 (Ref)	1.30	(1.20,	1.40)	
Regular physical activities	No	1 (Ref)	1.58	(1.56,	1.61)	0.374	1 (Ref)	1.56	(1.47,	1.66)	0.001
	Yes	1 (Ref)	1.55	(1.49,	1.62)		1 (Ref)	1.18	(1.01,	1.38)	
Obesity	No	1 (Ref)	1.57	(1.54,	1.60)	0.377	1 (Ref)	1.48	(1.38,	1.59)	0.489
	Yes	1 (Ref)	1.59	(1.55,	1.64)		1 (Ref)	1.54	(1.40,	1.69)	
Metabolic syndrome	No	1 (Ref)	1.58	(1.55,	1.60)	0.604	1 (Ref)	1.52	(1.43,	1.62)	0.275
	Yes	1 (Ref)	1.59	(1.53,	1.66)		1 (Ref)	1.40	(1.22,	1.61)	
DM	No	1 (Ref)	1.57	(1.54,	1.59)	0.039	1 (Ref)	1.50	(1.42,	1.59)	0.499
	Yes	1 (Ref)	1.70	(1.58,	1.84)		1 (Ref)	1.36	(1.01,	1.83)	
Hypertension^a^	No	1 (Ref)	1.57	(1.54,	1.60)	0.621	1 (Ref)	1.51	(1.42,	1.61)	0.285
	Yes	1 (Ref)	1.59	(1.52,	1.66)		1 (Ref)	1.38	(1.19,	1.61)	
Dyslipidemia^b^	No	1 (Ref)	1.57	(1.55,	1.60)	0.761	1 (Ref)	1.51	(1.43,	1.61)	0.289
	Yes	1 (Ref)	1.59	(1.51,	1.67)		1 (Ref)	1.36	(1.13,	1.64)	

All stratified analyses were adjusted for all covariates except for the variable used for stratification.

a No hypertension: SBP ≤ 120 mmHg and DBP ≤ 80 mmHg without medication/ ^b^No dyslipidemia: TC < 200 mg/dL without medication.

On the other hand, sex and comorbidity with DM significantly interacted with disability only in relation to depressive disorder, while engagement in regular physical activity emerged as a significant effect modifier only for anxiety disorder. Specifically, a more distinct increase in the HR for depressive disorder associated with disabilities was observed among young adults who are females or have comorbid DM, compared to those who are male or without DM, respectively. The increase in the risk for anxiety disorders associated with disabilities was greater among individuals who did not engage in regular physical activity (Table 3).

## Discussion

This study is the first to comprehensively investigate the association between disability and the risk of anxiety and depressive disorders among young adults aged 20 to 39. Disability was significantly associated with an increased risk of both conditions, and this association persisted even after adjusting for all covariates, including sociodemographic factors, health behaviours and comorbidities. An elevated risk of depressive disorders was consistently observed across most disability types with the highest increase observed in mental health-related disabilities. The risk of anxiety disorders also increased in approximately half of the disability types, with the greatest increase noted among those with epilepsy-related disabilities. Moreover, we found a more pronounced increase in the risk associated with disability among individuals who were younger, had lower income and did not smoke or drink alcohol. Several findings merit further attention.

First, previous studies that examined the entire population of individuals with disabilities across all age groups in Korea – among whom older adults constituted a substantial proportion – reported significantly lower likelihoods of depressive and anxiety disorder incidence in several disability types (e.g., brain injury-related disabilities, internal organ impairment-related disabilities) compared to individuals without disabilities (Lee *et al.*, 2023, 2025), interpreting these findings as potentially reflecting under-diagnosis, likely attributable to limited access to healthcare services among individuals with disabilities rather than evidence of genuinely lower incidence. On the other hand, the present study observed a consistently positive association between disability and the incidence of depressive and anxiety disorders across all disability types among young adults. This may reflect a lower degree of under-diagnosis in this younger population relative to older adults, potentially indicating a greater capacity to actively seek healthcare services, facilitated by more readily available social support to assist their health visits or more stable financial assistance compared to older individuals.

Second, while disabilities due to mental disorders exhibited the highest risk of depressive disorders, epilepsy emerged as the strongest risk factor for anxiety disorders among the various types of disabilities. The category classified as ‘disabilities due to mental disorders’ encompasses functional impairments arising from a broad spectrum of psychiatric conditions, including schizophrenia, bipolar disorder, organic mental disorders secondary to neurological brain damage and recurrent depressive disorders. Because individuals with preexisting depressive or anxiety disorders were excluded from the baseline population – thereby including only those newly diagnosed with depressive or anxiety disorders after the onset of their existing mental disorders – concerns about circularity between exposure and outcome were effectively minimised.

Although bipolar disorder is often initially misdiagnosed as major depressive disorder – since depressive episodes typically precede the recognition of hypomanic or manic episodes – it is uncommon for individuals already diagnosed with bipolar affective disorder to subsequently receive an additional diagnosis of depressive disorders. Such instances usually occur only when patients change healthcare providers and the new clinician, unaware of the prior bipolar disorder diagnosis, records depression instead (Stensland *et al.*, 2010). Therefore, it is highly unlikely that bipolar disorder substantially contributed to the elevated risk of depressive disorder observed among individuals with mental disability.

In contrast, other mental conditions causing disabilities, such as brain damage or schizophrenia, are well-documented risk factors for secondary depression. In schizophrenia, a depressive episode that develops after the remission or stabilisation of psychotic symptoms is clinically recognised as post-schizophrenic depression, clearly defined in the ICD-10 as F20.4 – ‘A depressive episode, which may be prolonged, arising in the aftermath of a schizophrenic illness’. (Guerrero-Jiménez *et al.*, 2022). Similarly, neurological brain damage, including traumatic brain injury and stroke, is frequently followed by the onset of depressive disorder, a phenomenon consistently reported as both common and clinically significant across psychiatric and neurological literature (Choi *et al.*, 2021; Conroy *et al.*, 2020; Dehbozorgi *et al.*, 2024).

Notably, the risk of anxiety disorder was most markedly elevated among individuals with epilepsy-related disabilities. Epilepsy, one of the most common chronic neurological conditions, is characterised by recurrent, unprovoked seizures resulting from transient disruptions in brain electrical activity (Batchelor and Taylor, 2021). Previous studies have highlighted the unique psychological challenges faced by young adults with epilepsy, including intense fear of seizure recurrence (Ryan and Räisänen, 2012) and threats to their sense of self, such as loss of self-esteem and sense of coherence (Gauffin *et al.*, 2010). These psychological burdens may contribute to the substantially elevated risk of anxiety disorders observed in this population. Pugh *et al.* (2005) demonstrated poorer mental health outcomes among young adults with epilepsy, compared to their middle-aged and older counterparts, despite relatively well-preserved physical function and activity levels, suggesting that older individuals generally cope better with epilepsy than middle-aged or younger adults do (Pugh *et al.*, 2005).

Intersectional subgroup analyses also revealed a few notable findings. First, it was found that females were more vulnerable to depressive disorders than males. It has been consistently reported that females are more prone to depression, despite higher suicide rate and greater prevalence of addictive behaviours in males (Ford and Erlinger, 2004). This female preponderance in rates of depression begins as early as adolescence (Cyranowski *et al.*, 2000). A few studies comprehensively reviewed risk factors leading to gender differences in depressive disorders, which include different coping styles and higher dependence on social support among females (Piccinelli and Wilkinson, 2000). Specifically, when stressful life events occur, males tend to distract themselves from their mood by engaging in physical or instrumental activities, whereas females more frequently employ self-consolatory strategies, such as ruminating over the possible causes and implications of their depression, thus prolonging the depressed mood (Hänninen and Aro, 1996). Additionally, since females have a stronger affiliative style than males, requiring greater social support for their psychological health, they may be more vulnerable to events affecting their close emotional ties, such as disabilities (Bebbington, 1998). How the gender disparities in disability-related depression can be translated into effective action needs to be given thoughtful consideration.

Second, within young adults, the risk of depressive and anxiety disorders associated with disability was higher among even younger individuals in their twenties compared to those in their thirties. The twenties represent a transitional stage during which individuals have recently emerged from adolescence and are in the process of establishing the foundations for adult life. Additionally, most individuals in their twenties are still engaged in educational pursuits (Settersten and Ray, 2010). Experiencing disabilities during this period may result in the loss of various opportunities for personal and professional development. Kwak *et al.* (2024) reported that Korean young adults with disabilities in their twenties are less likely to complete a college degree or pursue post-secondary education (Kwak *et al.*, 2024) and have significantly lower marriage rates compared to their non-disabled peers (Kwak *et al.*, 2024). These disparities may lead to greater psychological distress among younger individuals, who often hold higher expectations and aspirations for their future, yet may lack the life experience and coping capacity to manage significant adversity, than older populations.

Third, although income did not serve as a major confounder in the association between disability and the incidence of depressive or anxiety disorders, it appeared to act as an effect modifier. The heightened risk for depressive and anxiety disorders associated with disabilities was more pronounced among individuals in the low-income group compared to those in higher-income groups. Higher income may indicate that an individual is economically active and remains engaged in the labour market, which can foster a sense of self-esteem and self-efficacy, thereby contributing to better mental health outcomes. Furthermore, income can serve as a financial buffer, enabling access to activities and services that help alleviate the psychological burden associated with disability, such as hiring a caregiver, engaging in leisure or recreational activities, or participating in social networks. A follow-up in-depth study incorporating additional variables, such as employment status, occupational type, or social capital, is warranted to further elucidate the mechanisms underlying these associations.

Notably, individuals who engaged in health-promoting behaviours, such as abstaining from smoking and alcohol consumption, showed a more substantial increase in risk for both conditions compared to those who smoked and consumed alcohol. A cautious interpretation is necessary to explain this counterintuitive finding. Smoking and alcohol use are often linked with social networks and interactions, facilitating interpersonal bonding (Borsari and Carey, 2001; Christakis and Fowler, 2008). This is particularly relevant in the South Korean context, where smoking and drinking are not merely individual behaviours but are deeply embedded in social customs and cultural norms (Chun, 2012; Hyeonjin, 2012). These behaviours often serve as social lubricants in both professional and leisure settings, fostering intimacy and cohesion within communities, particularly among younger generations (Chun, 2012; Hyeonjin, 2012). As a result, not participating in smoking or drinking gatherings may compound feelings of exclusion or social isolation, which are already imposed by disabilities. Supporting this interpretation, one prior study conducted in Korea found that a slight increase in alcohol intake among individuals who were initially non-drinkers was associated with a reduced risk of depression, suggesting that, while heavy smoking and drinking are undoubtedly harmful, mild use during mentally challenging periods can serve as a coping mechanism or stress reliever (Cho *et al.*, 2024).

Finally, the risk of depressive symptoms associated with disabilities was higher among DM patients compared to those without DM. Diabetes in individuals in their 20s and 30s is more likely to be young-onset type 1 diabetes or maturity-onset diabetes of the young (MODY) (Song *et al.*, 2016; Tanenbaum and Gonzalez, 2012). Since type 1 DM has a higher prevalence of severe diabetic complications and shorter life spans, insulin therapy must be started immediately after the onset and continued throughout the lifetime, causing a serious physical, psychological and economic burden (Liese *et al.*, 2006). In this context, having DM and disability may pose a double burden to young populations.

Although this study makes an important contribution to the discourse on disability and mental health, several limitations should be acknowledged. First, the proportion of individuals with disabilities in our cohort was 1.5%, which is lower than estimates reported in other countries and slightly below the national prevalence among individuals aged 20–39 years during the corresponding period (2.0% in 2009–2010 and 1.9% in 2011–2012) (KWDI, 2025). This discrepancy may be explained by two factors: the narrow scope of disability criteria in Korea and a potential selection bias. In Korea, registration in the NDR requires assessment and certification by a qualified specialist physician based primarily on medical condition. This differs from the International Classification of Functioning, Disability, and Health (ICF) framework, which encompasses broader dimensions such as body functions, activity limitations, participation restrictions and environmental factors. Consequently, the scope of disability recognition in Korea is narrower, likely resulting in lower prevalence estimates compared with other countries that use ICF-based definitions. Additionally, our study population was drawn from the NHI Health Check-up database, which includes only individuals who participated in the health screening program. Individuals with limited access to these health check-ups – such as those with disabilities or mental health conditions – were therefore likely underrepresented, which may have led to an underestimation of both the prevalence of disability and the true risk of depressive and anxiety disorders among people with disabilities.

Second, it cannot be ruled out that the incidence of anxiety disorders may have been under-ascertained, partly due to clinicians’ diagnostic coding practices in combination with structural characteristics of the NHI reimbursement system. In clinical settings where anxiety and depressive disorders coexist, physicians often enter depressive disorder as the primary diagnosis and anxiety as a secondary one. However, it is typical that only the primary diagnosis is submitted to the NHI for reimbursement, as the NHI requires diagnostic codes that justify billed medical services rather than the full spectrum of a patient’s clinical conditions (Park *et al.*, 2017). Furthermore, since 2013, the reimbursement system has been revised to allow non-pharmacological consultations for mental health conditions to be reimbursed without requiring a psychiatric diagnosis code. Consequently, patients with mild or early-stage anxiety disorders – who frequently receive cognitive or counselling therapy – are less likely to have diagnostic codes recorded, whereas patients with depression, for whom pharmacological treatment remains the mainstay even in mild cases, are more frequently assigned diagnostic codes within the insurance database (Jang *et al.*, 2023; Ryu, 2013). Third, the presence of multiple disabilities could not be considered because the NHI service only retrieved information on the primary disability type from the NDR during data linkage. Additionally, information on the duration of disability was unavailable. These limitations may have resulted in either over-or under-estimation of the association observed across different disability types. Lastly, as the NHI database is originally designed for administrative and claims purposes, it provides limited information on socioeconomic characteristics. Consequently, only income level was included as a proxy for socioeconomic status, although mental health is likely influenced by a broader range of social determinants. Future studies incorporating a more comprehensive set of variables would offer a more in-depth understanding of the mental health challenges faced by young adults with disabilities.

## Conclusions

Our findings highlight that young adults with disabilities, a population that has been relatively overlooked in policy discussions, warrant greater attention. Certain types of disabilities that may not constitute major risks among older adults with disabilities in relation to mental conditions – such as epilepsy – may require closer monitoring and targeted intervention in young adults with disabilities. Future studies addressing the current limitations should be conducted to inform and guide policy efforts.

## Supporting information

10.1017/S204579602510036X.sm001 Lee et al. supplementary material 1 Lee et al. supplementary material

10.1017/S204579602510036X.sm002 Lee et al. supplementary material 2 Lee et al. supplementary material

## Data Availability

Data used in this study are available from the Korean National Health Insurance Service (http://nhiss.nhis.or.kr) under license and are not publicly accessible due to restrictions. However, they may be made available by the authors upon reasonable request and with permission from the KNHIS.

## Supplementary material

The supplementary material for this article can be found at https://doi.org/10.1017/S204579602510036X
