# Supplementary material for: Risk of depressive and anxiety disorders in young adults with disabilities: a nationwide cohort study in South Korea
Source: Epidemiol Psychiatr Sci. 2026 Jan 5;35:e2. doi: 10.1017/S204579602510036X (PMC12816932; doi:10.1017/S204579602510036X)
Supplement: Lee et al. supplementary material 2 — Lee et al. supplementary material [file S204579602510036Xsup002.docx]

**Supplementary Methods**

Data sources

The Republic of Korea has a single-payer nationwide social health insurance system in which all residents are enrolled mandatorily.^1^ The National Health Insurance Database (NHID), managed by the National Health Insurance Service (NHIS), includes all information on health service utilization claimed by healthcare providers for reimbursement purposes, including diagnoses, prescriptions, and healthcare costs. Additionally, the NHI provides a biennial health checkup program free of charge to all citizens over 40 years and to all formal workers (including self-employees) regardless of age.^2^ The information collected during these checkups is recorded in the Health Checkup Database.

As of 2020, the National Disability Registry (NDR) covered 99.9% of the total estimated population with disabilities in the Republic of Korea.^3^ While the concept of disability extends beyond mere clinical impairment,^4^ disabilities in the NDR are defined strictly based on medical conditions. Registration in the NDR requires an official disability certificate issued by a qualified specialist physician in the relevant field.^5^

Table S1. Definitions of covariates.

| Covariates | Definition |
| --- | --- |
| Income | The NHI database provides income information in 20 insurance premium quantiles, rather than specific monetary values. The study sample was categorized into five income groups: MA beneficiaries (representing the lowest income group), and the remaining NHI enrollees grouped into quartiles based on their premium quantiles. A low-income group was constructed by combining MA beneficiaries with the 1^st^ quartile of NHI enrollees. |
| Obesity | BMI: ≥25 kg/m^2^ |
| Metabolic syndrome | Presence of ≥3 of the following components:  (1) WC ≥ 85 cm for women and ≥ 90 cm for men  (2) [SBP](https://www.sciencedirect.com/topics/medicine-and-dentistry/systolic-blood-pressure) ≥ 130 mmHg, [DBP](https://www.sciencedirect.com/topics/medicine-and-dentistry/diastolic-blood-pressure) ≥ 85 mmHg, or identified hypertension  (3) FBG ≥ 100 mg/dL or identified DM  (4) HDL < 50 mg/dL for women, < 40 mg/dL for men, or identified [dyslipidemia](https://www.sciencedirect.com/topics/medicine-and-dentistry/dyslipidemia)  (5) TC ≥150 mg/dL or identified [dyslipidemia](https://www.sciencedirect.com/topics/pharmacology-toxicology-and-pharmaceutical-science/dyslipidemia) |
| DM | FBG ≥ 126 mg/dL or patients with DM medications & E11-E14 |
| Hypertension | SBP ≥ 140mmHg or DBP ≥ 90mmHg or patients with hypertension medications & I10-13, I15 |
| Dyslipidemia | TC ≥ 240mg/dL or patients with dyslipidemia medications & E78 |

BMI: body mass index (kg/m^2^) , WC: waist circumference , SBP: systolic blood pressure, DPB: diastolic blood pressure , FBG: fasting blood glucose, HDL: high-density lipoprotein, MA: Medical Aid, TC: triglyceride, DM: diabetes mellitus.

Table S2. Study sample characteristics (n, %).

| Categories | | | With disability, total | Mild | Severe | P |
| --- | --- | --- | --- | --- | --- | --- |
|  |  |  | 87889 | 58185 | 29704 |  |
| Sociodemographic  characteristics | Sex–Male: n (%) | | 71,261 (81.1) | 49821 (85.6) | 21440 (72.2) | < .0001 |
|  | Age (mean ± SD*) | | 32.33 ± 4.96 | 32.3 ± 5.0 | 31.0 ± 5.5 | < .0001 |
|  | Age group: n (%) | 20–29 years | 25062 (28.5) | 13495 (23.2) | 11567 (39.0) | < .0001 |
|  |  | 30–39 years | 62827 (71.5) | 44690 (76.8) | 18137 (61.1) |  |
|  | Income level–low: n (%) | | 29559(33.6) | 13106 (22.5) | 16453 (55.4) | < .0001 |
| Health behavior: n (%) | Currently smoking | | 36,357 (41.4) | 29073 (50.0) | 7284 (24.5) | < .0001 |
|  | Currently drinking alcohol | | 50,306 (57.2) | 39314 (67.6) | 10992 (37.0) | < .0001 |
|  | Currently exercising regularly (walking, jogging, cycling etc) | | 13,802 (15.7) | 9523 (16.4) | 4279 (14.4) | < .0001 |
| Comorbidities: n (%) | Obesity | | 31,918 (36.3) | 22922 (39.4) | 8996 (30.3) | < .0001 |
|  | Metabolic syndrome | | 15,219 (17.3) | 11012 (18.9) | 4207 (14.2) | < .0001 |
|  | Diabetes | | 3,308 (3.6) | 2232 (3.8) | 1076 (3.6) | < .0001 |
|  | Hypertension | | 11,468 (13.1) | 7742 (13.3) | 3726 (12.5) | < .0001 |
|  | Dyslipidemia | | 8,146 (9.3) | 6006 (10.3) | 2140 (7.2) | < .0001 |
| Biometrics: Mean ± SD | Height, cm | | 169.2 ± 9.2 | 170.8 ± 8.2 | 166.1 ± 10.2 | < .0001 |
|  | Weight, kg | | 68.9 ± 14.1 | 71.1 ± 13.6 | 64.5 ± 14.1 | < .0001 |
|  | BMI, kg/m2 | | 23.9 ± 3.9 | 24.3 ± 3.7 | 23.3 ± 4.1 | < .0001 |
|  | Waist circumference, cm | | 81.0 ± 9.9 | 81.9 ± 9. 6 | 79.2 ± 10.4 | < .0001 |
|  | Fasting glucose, mg/dL | | 93.4 ± 21.0 | 94.1±21.2 | 92.0 ± 20.7 | < .0001 |
|  | Systolic BP, mmHg | | 120.8 ± 13.6 | 121. 7±13.3 | 119.1 ± 14.1 | < .0001 |
|  | Diastolic BP, mmHg | | 75.9 ± 9.75 | 76.4 ± 9.6 | 74.8 ± 9.9 | < .0001 |
|  | Total cholesterol, mg/dL | | 186.6 ± 35.7 | 190.0 ± 35.4 | 179.0 ± 35.2 | < .0001 |
|  | HDL-C, mg/dL | | 54 ± 20.4 | 54.0 ± 21.4 | 53.9 ± 18.2 | < .0001 |
|  | LDL-C, mg/dL | | 106.4 ± 35.8 | 108.1 ± 35.5 | 103.1 ± 36.1 | < .0001 |
|  | Triglyceride, mg/dL (min–max) | | 111.5 (111.1–112.0) | 119.1 (118.5–119.7) | 98.1 (97.4–98.7) | < .0001 |
| Outcome | | | | | | |
| Incidence of outcome: n (%) | Depression | | 14,865 (16.9) | 9041 (15.5) | 5824 (19.6) | < .0001 |
|  | Anxiety | | 1,233 (1.4) | 736 (1.3) | 497 (1.7) | < .0001 |
| Follow-up duration |  | |  |  |  |  |
| Mean ± SD | Depression | | 10.4 ± 2.8 | 10.5 ± 2.7 | 10.0 ± 3.0 | < .0001 |
|  | Anxiety | | 11.2 ± 1.5 | 11.5 (10.0–12.2) | 11.0 (9.3–12.2) | < .0001 |
| Median (Q1–Q3) | Depression | | 11.3 (9.7–12.2) | 11.3 ± 1. 5 | 11.0 ±1.7 | <. 0001 |
|  | Anxiety | | 11.7 (10.4–12.2) | 12 (10.7–12.3) | 11.4 (10.0–12.2) | <. 0001 |

*SD: standard deviation

Table S3. Association between disability and depressive and anxiety disorders from sequential models with incremental adjustment for covariates

| Disability  indicators | Categories | Depressive disorders | | | | | | | | | Anxiety disorders | | | | | | | | |
| --- | --- | --- | --- | --- | --- | --- | --- | --- | --- | --- | --- | --- | --- | --- | --- | --- | --- | --- | --- |
|  |  | M1 | | | M2 | | | M3 | | | M1 | | | M2 | | | M3 | | |
|  |  | HR (95% CI) | | | HR (95% CI) | | | HR (95% CI) | | | HR (95% CI) | | | HR (95% CI) | | | HR (95% CI) | | |
| Disability status^**^ | No disability | 1 (Ref.) | | | 1 (Ref.) | | | 1 (Ref.) | | | 1 (Ref.) | | | 1 (Ref.) | | | 1 (Ref.) | | |
|  | Yes | 1.47 | (1.44, | 1.49) | 1.58 | (1.56, | 1.61) | 1.58 | (1.55, | 1.60) | 1.43 | (1.35, | 1.51) | 1.48 | (1.40, | 1.57) | 1.50 | (1.42, | 1.59) |
| Disability severity^**^ | No disability | 1 (Ref.) | | | 1 (Ref.) | | | 1 (Ref.) | | | 1 (Ref.) | | | 1 (Ref.) | | | 1 (Ref.) | | |
|  | Mild (Grade 4-6) | 1.32 | (1.29, | 1.35) | 1.44 | (1.41, | 1.47) | 1.42 | (1.39, | 1.45) | 1.27 | (1.18, | 1.36) | 1.32 | (1.23, | 1.42) | 1.32 | (1.23, | 1.42) |
|  | Severe (Grade 1-3) | 1.77 | (1.73, | 1.82) | 1.86 | (1.82, | 1.91) | 1.88 | (1.84, | 1.93) | 1.74 | (1.59, | 1.90) | 1.79 | (1.64, | 1.95) | 1.87 | (1.71, | 2.04) |
| Disability type^**^ | No disability | 1 (Ref.) | | | 1 (Ref.) | | | 1 (Ref.) | | | 1 (Ref.) | | | 1 (Ref.) | | | 1 (Ref.) | | |
|  | Disabilities in extremities | 1.35 | (1.32, | 1.38) | 1.47 | (1.44, | 1.50) | 1.45 | (1.42, | 1.49) | 1.28 | (1.18, | 1.38) | 1.33 | (1.23, | 1.44) | 1.33 | (1.23, | 1.44) |
|  | Disability due to brain damage | 1.89 | (1.74, | 2.06) | 2.00 | (1.84, | 2.17) | 2.02 | (1.86, | 2.19) | 2.47 | (1.93, | 3.15) | 2.54 | (1.99, | 3.24) | 2.62 | (2.05, | 3.35) |
|  | Visual disability | 1.24 | (1.18, | 1.30) | 1.33 | (1.27, | 1.40) | 1.33 | (1.27, | 1.39) | 1.07 | (0.89, | 1.28) | 1.11 | (0.93, | 1.33) | 1.12 | (0.93, | 1.34) |
|  | Hearing disability | 1.25 | (1.17, | 1.33) | 1.29 | (1.21, | 1.37) | 1.30 | (1.22, | 1.38) | 0.95 | (0.74, | 1.22) | 0.97 | (0.76, | 1.24) | 0.99 | (0.77, | 1.26) |
|  | Speech and language disability | 1.40 | (1.20, | 1.64) | 1.51 | (1.30, | 1.77) | 1.54 | (1.32, | 1.79) | 2.16 | (1.41, | 3.32) | 2.25 | (1.47, | 3.45) | 2.33 | (1.52, | 3.57) |
|  | Intellectual disability | 1.87 | (1.79, | 1.95) | 2.00 | (1.92, | 2.09) | 2.05 | (1.96, | 2.14) | 1.92 | (1.67, | 2.21) | 2.01 | (1.75, | 2.31) | 2.18 | (1.89, | 2.51) |
|  | Disability due to autism | 1.74 | (1.38, | 2.20) | 2.17 | (1.71, | 2.74) | 2.27 | (1.79, | 2.87) | 0.57 | (0.14, | 2.28) | 0.66 | (0.16, | 2.62) | 0.73 | (0.18, | 2.92) |
|  | Disability due to mental disorders | 5.06 | (4.69, | 5.45) | 5.00 | (4.64, | 5.39) | 4.98 | (4.62, | 5.37) | 3.11 | (2.32, | 4.18) | 3.08 | (2.29, | 4.14) | 3.26 | (2.42, | 4.38) |
|  | Disability due to renal failure | 1.53 | (1.33, | 1.76) | 1.55 | (1.35, | 1.78) | 1.57 | (1.36, | 1.80) | 2.44 | (1.67, | 3.55) | 2.44 | (1.68, | 3.56) | 2.48 | (1.70, | 3.61) |
|  | Disability due to heart conditions | 1.62 | (1.19, | 2.20) | 1.66 | (1.23, | 2.26) | 1.68 | (1.24, | 2.28) | 0.91 | (0.23, | 3.64) | 0.92 | (0.23, | 3.69) | 0.94 | (0.23, | 3.75) |
|  | Disability due to respiratory conditions | 1.50 | (0.81, | 2.78) | 1.49 | (0.80, | 2.77) | 1.54 | (0.83, | 2.86) | . |  |  | . |  |  | . |  |  |
|  | Disability due to liver disease | 1.49 | (0.88, | 2.51) | 1.52 | (0.90, | 2.57) | 1.56 | (0.93, | 2.64) | 2.51 | (0.63, | 10.02) | 2.53 | (0.63, | 10.13) | 2.66 | (0.67, | 10.63) |
|  | Facial deformities | 1.46 | (1.11, | 1.92) | 1.54 | (1.17, | 2.02) | 1.54 | (1.17, | 2.02) | 0.99 | (0.32, | 3.08) | 1.02 | (0.33, | 3.16) | 1.03 | (0.33, | 3.20) |
|  | Disability due to ostomy | 1.68 | (1.22, | 2.31) | 1.83 | (1.33, | 2.52) | 1.85 | (1.34, | 2.54) | 0.52 | (0.07, | 3.65) | 0.54 | (0.08, | 3.82) | 0.55 | (0.08, | 3.87) |
|  | Disability due to epilepsy | 3.61 | (3.04, | 4.30) | 3.80 | (3.19, | 4.51) | 3.82 | (3.21, | 4.54) | 11.27 | (8.17, | 15.56) | 11.51 | (8.34, | 15.88) | 12.05 | (8.73, | 16.63) |

M- unadjusted ; M2- adjusted for age and sex; M3-adjusted for age and sex, income level, smoking status, drinking status, regular physical activity, obesity, and metabolic syndrome

^**^Separate analyses were conducted for disability status, severity, and type.

**References**

1. Cheol Seong S, Kim Y-Y, Khang Y-H, et al. Data resource profile: The National Health Information Database of the National Health Insurance Service in South Korea. *International Journal of Epidemiology* 2017; **46**(3): 799-800.

2. Shin DW, Cho J, Park JH, Cho B. National General Health Screening Program in Korea: History, current status, and future direction. *Precision and Future Medicine* 2022; **6**(1): 9-31.

3. KSIS. Estimated number of disabled people and disability registration rate: <https://kosis.kr/statHtml/statHtml.do?orgId=117&tblId=DT_11732S0103&conn_path=I2> (Accessed 5 February 2024). Korean Statistical Information Service.

4. World Health Organization. International Classification of Functioning, Disability, and Health: Children & Youth Version: ICF-CY: World Health Organization; 2007.

5. Kim M, Jung W, Kim SY, Park JH, Shin DW. The Korea National Disability Registration System. *Epidemiology and Health* 2023: e2023053.
